# Detection and homology analysis of carbapenem resistant Acinetobacter baumannii resistance gene

**DOI:** 10.3389/fcimb.2022.987260

**Published:** 2023-01-06

**Authors:** Hua-Liang Huang, Yue-Yu Li, Hong-Bo Guo

**Affiliations:** Department of Laboratory, Inner Mongolia Baogang Hospital, Baotou, China

**Keywords:** carbapenem resistant Acinetobacter baumannii, drug resistance gene, pulsed field gel electrophoresis, homology, infection microbiology

## Abstract

**Objective:**

To explore the carrying status and homology of carbapenem resistant Acinetobacter baumannii (CRAB) in our hospital.

**Methods:**

From January 2015 to December 2017, 52 strains of acinetobacter baumannii isolated from the bacteria room of the clinical laboratory of Baogang hospital in Inner Mongolia were selected as the research object. K-B disk diffusion method and Vitek-2 were used to determine the drug sensitivity of Acinetobacter baumannii. The drug resistance gene was detected by polymerase chain reaction (PCR) and its homology was analyzed by pulsed field gel electrophoresis (PFGE).

**Results:**

Except for Cefoperazone/sulbactam, other antibiotics were resistant to ab. The detection rate of drug resistance gene class C β-lactamases (ADC) was 100%, and the higher detection rates of other drug resistance genes were class D β-lactamases (OXA)-51 (36 strains, 90.0%),disinfectant gene qacE△1-sull (32 strains, 80.0%), and klebsiella pneumoniae carbapenemase (KPC) gene was not detected. 2-8 drug resistance genes were detected in each CRAB strain, and the strains with 6 drug resistance genes were the most (15 strains, 37.5%); Among the detected drug-resistant gene combinations, ADC+OXA-23 + OXA-51 gene was detected at the same time (29 strains, 72.5%), followed by ADC+ intl1 + qacE △ 1-sull gene (26 strains, 65.0%), ADC + qacE △ 1-sull + ant (3 ‘‘) -i gene (19 strains, 47.5%), and 11 strains (27.5%). There were 19 different types in PFGE homology test, each type was 1-9 strains, including 9 strains of A5 type and 8 strains of A18 type, mainly from intensive care unit.

**Conclusion:**

CRAB in the hospital is highly resistant to common clinical antibiotics. OXA-23 and OXA-51 genes are most likely to be the main factors causing drug resistance of Acinetobacter baumannii in the hospital. Homology analysis showed that there was CRAB nosocomial infection transmission in different wards of the hospital.

## Introduction

Acinetobacter baumannii (AB) is a conditional pathogen (Yuxing and Hong, 2007), accounting for more than 80% of clinically isolated Acinetobacter. It widely exists in hospital environment and nature (Zhizhi et al., 2017). Due to the abuse of antibiotics, the continuous variation of bacteria and the timeliness of the research and development of new antibiotics, the number of effective drugs that can be used for multidrug-resistant AB continues to decrease (Chen et al., 2015). From 1993 to 2004, AB resistance in the United States increased 10 times (Lockhart et al., 2007). In China, AB resistance also increased from 31% ~ 39% in 2005 to 62.4% ~ 66.7% in 2014 (Hu et al., 2016). AB infection has become an increasingly prominent public health problem (Chen et al., 2015) and the main threat of global nosocomial infection (Anunnatsiri and Tonsawan, 2011). Imipenem (IPM), belonging to carbapenems, is a kind of broad-spectrum and efficient antibiotics β− Lactam antibiotics have strong antibacterial activity against AB, but with the wide clinical application, the drug resistance rate is increasing year by year (Yanan et al., 2021).Carbapenem resistant Acinetobacter baumannii (CRAB) refers to ab that is resistant to carbapenem antibiotics such as imipenem or meropenem (Chen et al., 2015). At present, it is considered that carbapenemase production is the main reason for ab’s resistance to carbapenem antibiotics (Yanan et al., 2021).

The drug resistance mechanism of AB is very complex and can be mediated by a variety of drug resistance mechanisms. If the expression of outer membrane protein changes, it can cause the barrier of antibiotic permeability; Changes of topoisomerase and DNA gyrase; Overexpression of efflux pump of various drugs (Abdi et al., 2020). This paper will analyze the drug resistance characteristics and main epidemic types of Acinetobacter baumannii in our hospital, so as to provide basis for clinical better selection of antibiotics and control of nosocomial infection. This paper studies the homology and drug resistance mechanism of drug-resistant CRAB, which is reported as follows.

## Materials and methods

### Strain source

40 CRAB strains isolated from the bacteria room of the clinical laboratory of Inner Mongolia Baogang hospital from January 2015 to December 2017 were selected as the research objects. Remove duplicate strains isolated from the same patient. The isolated strains were identified and tested by automatic microbial identification and drug sensitivity instrument. Some drug sensitivity tests and reviews were conducted by disk diffusion method (K-B method). The drug sensitivity results were judged according to the American Society for clinical laboratory standardization (CLSI) version 2021. The quality control strains were Escherichia coli ATCC25922, Pseudomonas aeruginosa atcc27853, enzyme producing Escherichia coli atcc35218 and Klebsiella pneumoniae atcc70060.

### Main instruments and reagents

PCR FQZD (BIOER), nucleic acid electrophoresis apparatus (DYY-6C, Beijing 61), automatic gel imaging analysis system (ZF-258, Shanghai Jia Peng), fluorescence quantitative polymerase chain reaction (PCR) instrument (FQD-48A, BIOER company), electronic constant temperature stainless steel water bath pot (HHS-2S, Shanghai Yichang instrument).

RNA extraction reagent and DNA loading buffer were purchased from cwbio company. Dnamarker DL2000, reverse transcription PCR kit and Supermix were purchased from tran company. Agarose and dyes were purchased from xhly company.

### Antimicrobial susceptibility test

The susceptibility of the strains to 15 kinds of antibiotics was detected by disk diffusion method (K-B method). Antibacterial drugs include: Cefoperazone/sulbactam, cefotaxime, ceftriaxone, levoflOXAcin, tobramycin, ceftazidime, imipenem, meropenem, piperacillin, piperacillin/tazobactam, gentamicin, amikacin, cefepime, minocycline, compound minocycline, etc. The pieces of paper were purchased from oxid. Columbia blood tablet was purchased from French merier company, and MH tablet was purchased from Zhengzhou Beiruite company. The judgment of drug sensitivity results shall be carried out according to the 2021 standard of American Society for clinical laboratory standardization (CLSI) (Humphries et al., 2021).

### Homology analysis of strains

PFGE homology detection: after CRAB was isolated and cultured, the bacteria suspension with OD value of 3.6 ~ 4.0 was adjusted by turbidimeter. Add 1%seakem gold SDS to prepare gel. Digest with protease K, wash with pure water for 2 times, and then wash with TE for 4 times, about 15min each time. ApaI endonuclease was used for enzyme digestion and incubated in 37°C water bath for 4H. PFGE was performed in a pulsed field gel electrophoresis apparatus. The electrophoretic parameters were 5 ~ 20s, 14°C, 120° pulse angle electrophoresis for 19h. After electrophoresis, nucleic acid staining was performed, which was put into a gel apparatus for observation and photo preservation.

### Gene detection of CRAB

Eight strains of multidrug-resistant bacteria were selected as the drug-resistant group, and the gene expression levels of the strains in the drug-resistant group and the sensitive group were detected.

Extract the strain RNA according to the operation steps of the bacterial RNA extraction kit of Omega company, use the nucleic acid protein analyzer to detect the concentration of RNA and carry out electrophoresis. The genes were amplified by realtime PCR. The reaction system is 20ul, including 0.4ul of upstream and downstream primers and 5 UL of cDNA template, 2 × PerfectStartTM Green qPCR Super Mix10uL. Make up the volume to 20ul with DD H2O. The amplification conditions were three-step, the first step was 95 °C, 30s; 1 cycle; The second step is 95 °C, 5S; 60 °C, 30s, 40 cycles. See **Table 1**.

**Table 1:** Gene primer sequence and product size of realtime PCR.

Gene name	Sequence
adeB-F	TTAACGATAGCGTTGTAACC
adeB-R	TGAGCAGACAATGGAATAGT
adeJ-F	GATTCAGCGTGGTATGGC
adeJ-R	ACGTTCGAGACGTGGAGA
abeG-F	GTAGGTGTAGGCTTATGCA
abeG-R	GTACCGAAGTGACTGAAAT
adeR-F	ATGTTTGATCATTCTTTTCTTTTG
adeR-R	TTAATTAACATTTGAAATATG
adeS-F	ATGAAAAGTAAGTTAGGAATTAGTAAG
adeS-R	TTAGTTATTCATAGAAATTTTATG
qadeB-F	AACGGACGACCATCTTTGAGTATT
qadeB-R	CAGTTGTTCCATTTCACGCATT
qadeJ-F	ATGAGAAACTGATTGCAGCTC
qadeJ-R	TGAGGAGTATCTTCCTGACCA
qabeG-F	AGGCTTCGGCTTATCGAAAC
qabeG-R	AGAGGGCTAAGCACCAATGG
16sRNA- F	CAGCTCGTGTCGTGAGATGT
16sRNA- R	CGTAAGGGCCATGATGACTT

### Data analysis

Import the images saved by the gel imager into the bionumerily software for processing and analysis, and calculate the similarity coefficient between the strains with the Dice coefficient (Humphries et al., 2021). SD=2nxy/(nx+ny). Where nx represents the total number of bands of strain x, ny represents the total number of bands of strain y, nxy is the number of bands common to strain xy, and SD reflects the similarity of strains. The range is 0-1. 0 means completely different and 1 means exactly the same. The similarity coefficient of 80% is the typing boundary value, the similarity ≥ 80% is the same subtype, and the similarity < 80% is different genotypes.

## Result

### Clinical distribution and drug resistance of multidrug resistant AB

A total of 40 CRAB strains were collected from 40 patients. The sources of specimens were vascular catheter tip (9 strains), drainage fluid (5 strains), blood (5 strains), bronchial lavage fluid (5 strains), urine (4 strains), secretion (4 strains), sputum (4 strains), cerebrospinal fluid (2 strains), ascites (1 strain), bile (1 strain), and 28 strains of CRAB were from intensive care unit (ICU). See **Table 2**.

**Table 2:** Drug resistance spectrum of 40 crabs.

Strain number	Source department	PFGE number	Drug resistance
1	Microsurgery Department	A18	SAM CTX MEM CRO FEP IPM GEN CIP LVX
2	Microsurgery Department	A9	CTX CAZ MEM CRO FEP IPM GEN TOP LVX CIP SXT
3	Pediatric intensive care unit	A15	CSL TZP CRO FEP IPM GEN LVX CIP SXT
4	ICU	A5	CSL SAM MEM TZP CRO FEP IPM GEN TOP LVX CIP
5	ICU	A6	CSL SAM MEM TZP CRO FEP IPM TOP GEN AMK LVX CIP
6	ICU	A17	SAM MEM MNO TZP CRO FEP IPM GEN LVX CIP
7	ICU	A9	PIP TZP CAZ CRO FEP IPM MEM AMK GEN TOP CIP LVX SXT
8	ICU	A18	CSL SAM MEM MNO TZP CRO FEP IPM GEN LVX CIP
9	Hepatology Department	A3	SAM MEM POL MNO TZP CRO CRO IPM TOP GEN LVX CIP
10	Respiratory intensive care unit	A5	SAM MEM MNO TZP CRO FEP IPM TOP AMK GEN LVX CIP
11	Pancreatic surgery	A16	SAM MEM MNO TZP CRO FEP IPM LVX CIP
12	Respiratory intensive care unit	A5	SAM MEM MNO TZP CRO FEP IPM TOP GEN LVX CIP
13	Respiratory intensive care unit	A5	SAM MEM MNO TZP CRO FEP IPM TOP GEN LVX CIP
14	ICU	A17	SAM MEM MNO CRO FEP IPM GEN LVX CIP
15	Pancreatic surgery	A23	SAM MEM MNO CRO FEP IPM GEN CIP LVX
16	ICU	A5	SAM MEM MNO TZP CRO FEP IPM GEN TOP CIP LVX
17	Respiratory intensive care unit	A18	TZP CRO FEP MEM IPM GEN LVX CIP SXT MNO
18	Three blood departments	A1	SAM TZP FEP MEM IPM CIP SXT CSL
19	Nephrology	A12	FEP MEM IPM GEN LVX CIP SXT
20	Respiratory intensive care unit	A18	SAM TZP CRO FEP MEM IPM GEN LVX CIP MNO CSL
21	Respiratory intensive care unit	A14	PIP SAM TZP CAZ CRO FEP MEM IPM GEN LVX CIP
22	Burn department	A11	IPM AMK GEN TOB
23	ICU	A13	PIP SAM TZP CAZ CRO FEP IPM MEM TOP AMK GEN CIP LVX
24	Neurosurgery	A8	CSL SAM MEM TZP CRO FEP IPM GEN TOP LVX CIP
25	Neonatal ICU	A9	MEM TZP CRO FEP IPM AMK TOB GEN CIP LVX SXT
26	Pediatric intensive care unit	A2	CRO FEP IPM MEM AMK TOB GEN LVX CIP SXT TGC
27	ICU	A19	MEM IPM GEN LVX CIP SXT
28	ICU	A5	TZP CRO FEP MEM IPM AMK TOP GEN LVX CIP
29	Hepatobiliary echinococcidae	A18	TZP CRO FEP MEM IPM GEN LVX CIP
30	ICU	A10	CRO FEP MEM IPM GEN TOP LVX CIP SXT
31	ICU	A4	SAM CRO FEP CTX MEM IPM GEN TOP CIP LVX MNO
32	Emergency intensive care unit	A5	TZP CRO FEP MEM IPM AMK TOP GEN LVX CIP
33	Infection intensive care unit	A5	SAM MEM TZP CRO FEP IPM GEN TOB LVX CIP
34	General surgery	A5	SAM MEM MNO CRO FEP IPM GEN TOB LVX CIP SXT
35	Pediatric intensive care unit	A18	SAM MEM MNO TZP CRO FEP IPM GEN LVX CIP
36	Pediatric intensive care unit	A7	TZP CRO FEP MEM IPM TOB GEN CIP LVX
37	ICU	A18	TZP CRO FEP IPM MEM GEN LVX CIP
38	Pediatric intensive care unit	A18	MEM MNO TZP CRO FEP GEN LVX CIP SXT
39	ICU	A9	MEM TZP CRO FEP IPM AMK TOB GEN CIP LVX SXT CIP
40	Pediatric internal medicine department I	A9	SAM MEM MNO TZP CRO FEP IPM GEN TOB AMK LVX CIP

Among them, 40 AB strains were resistant to cefotaxime, ceftriaxone, levoflOXAcin, tobramycin, ceftazidime, imipenem, meropenem, piperacillin, piperacillin/tazobactam, gentamicin, amikacin, cefepime and minocycline, and the resistance rate was 100%. 10 AB strains were sensitive to Cefoperazone/sulbactam, and the resistance rate was 25.0%. 36 AB strains were sensitive to cotrimOXAzole, and the resistance rate was 90.4%. See **Table 3**.

**Table 3:** Resistance of strains of CRAB resistant bacteria to common antibiotics.

Antibiotics	Number of resistant strains	Drug resistance rate (%)
Cefoperazone/sulbactam	10	25.0
Cefatriaxone	40	100
Levofloxacin	40	100
Tobramycin	40	100
Ceftazidime	40	100
Imipenem	40	100
Meropenem	40	100
Piperacillin	40	100
Piperacillin/tazobactam	40	100
Gentamicin	40	100
Amikacin	40	100
Cefepime	40	100
Minocycline	40	100
Compound sulfamethoxazole	36	90.4

### Test results of main drug resistance genes

**Figure 1:**
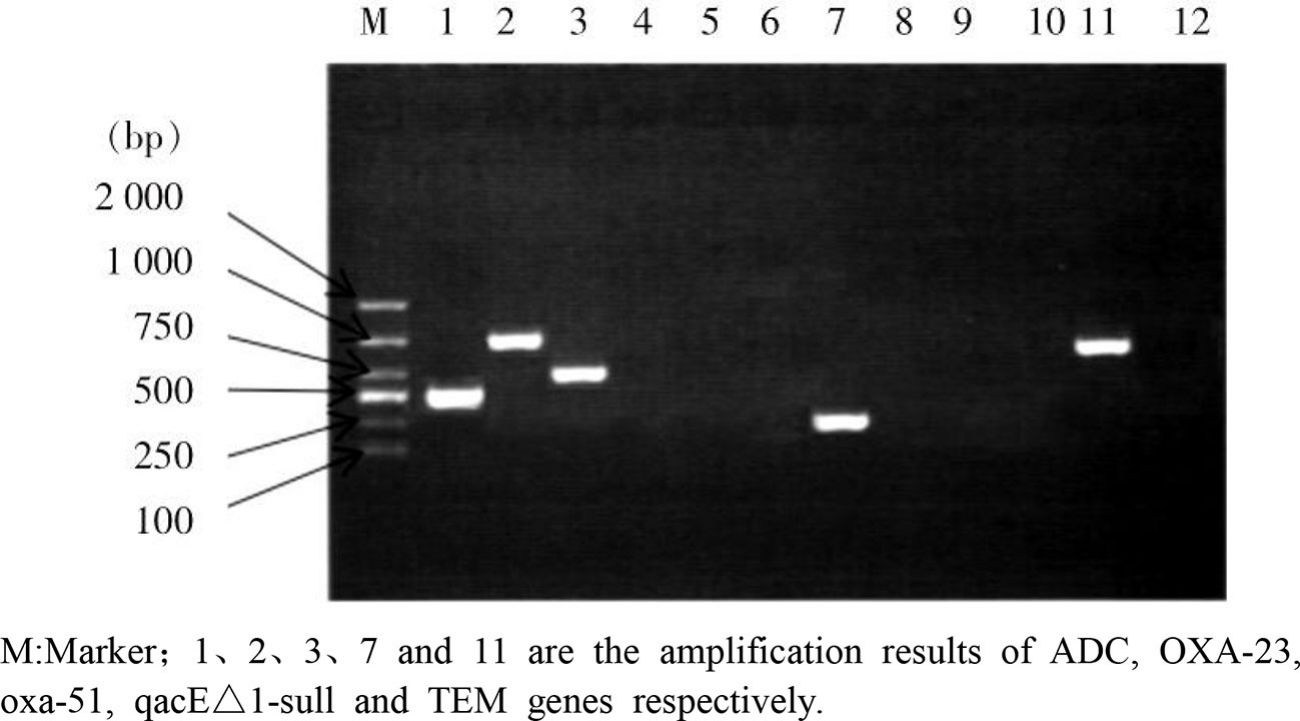
PCR amplification map of some drug resistance genes. M:Marker;1、2、3、7 and 11 are the amplification results of ADC, OXA-23, oxa-51, qacE△1-sull and TEM genes respectively.

ADC gene was detected in all 40 CRABs. The detection rates of other drug resistance genes were OXA-51 (90.0%), qacE△1-sull (80.0%), OXA-23 (77.5%), intl1 (72.5%), and KPC gene was not detected. 2-8 drug resistance genes were detected in each CRAB strain, and the strains with 6 drug resistance genes were the most (37.5%); Among the drug-resistant gene combinations detected, ADC + OXA-23 + OXA-51 gene was the most detected at the same time (29 strains, 72.5%), followed by ADC + intl1 + qacE△1-sul1 gene (26 strains, 65.0%), ADC + qacE△1-sull + ant (3″) -1 gene (19 strains, 47.5%), ADC + OXA-23 + OXA-51 + intl1 + qacE△1-sull gene (18 strains, 45.0%), and 11 strains (27.5%). ADC+ant (3″) -i + ACC(3) -i gene was detected. See **Figure 1** and **Table 4**.

**Table 4:** Detection of main drug resistance genes of 40 crab strains.

Drug resistance gene	N	Rate (%)
ADC	40	100.0
OXA-51	36	90.0
qacE△1-sull	32	80.0
OXA-23	31	77.5
Intl1	29	72.5
ant (3”)-I	23	57.5
TEM	23	57.5
aac (3’’)-I	14	35.5
tnpu	6	15.5
traA	3	7.5
CTX-M-9	1	2.5

ADC, Class C β-lactamases; OXA, Class D β-lactamases; qacE△1-sull, Disinfectant gene; Intl1,Integron resistance gene; ant (3”)-I 、aac (3”)-I,aminoglycoside resistance gene; TEM、CTX-M-9, Class A β-lactamases; tnpu,Transposon resistance gene;traA,Conjugated plasmid resistance gene.

### PFGE clustering tree and drug resistance spectrum of 40 strains

**Figure 2:**
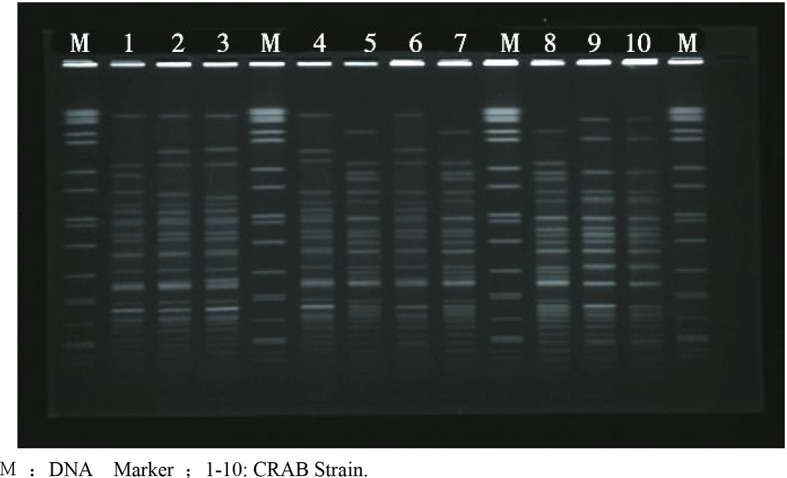
PFGE electrophoresis patterns of some crab strains. M: DNA Marker ;1-10: CRAB Strain.

**Figure 3:**
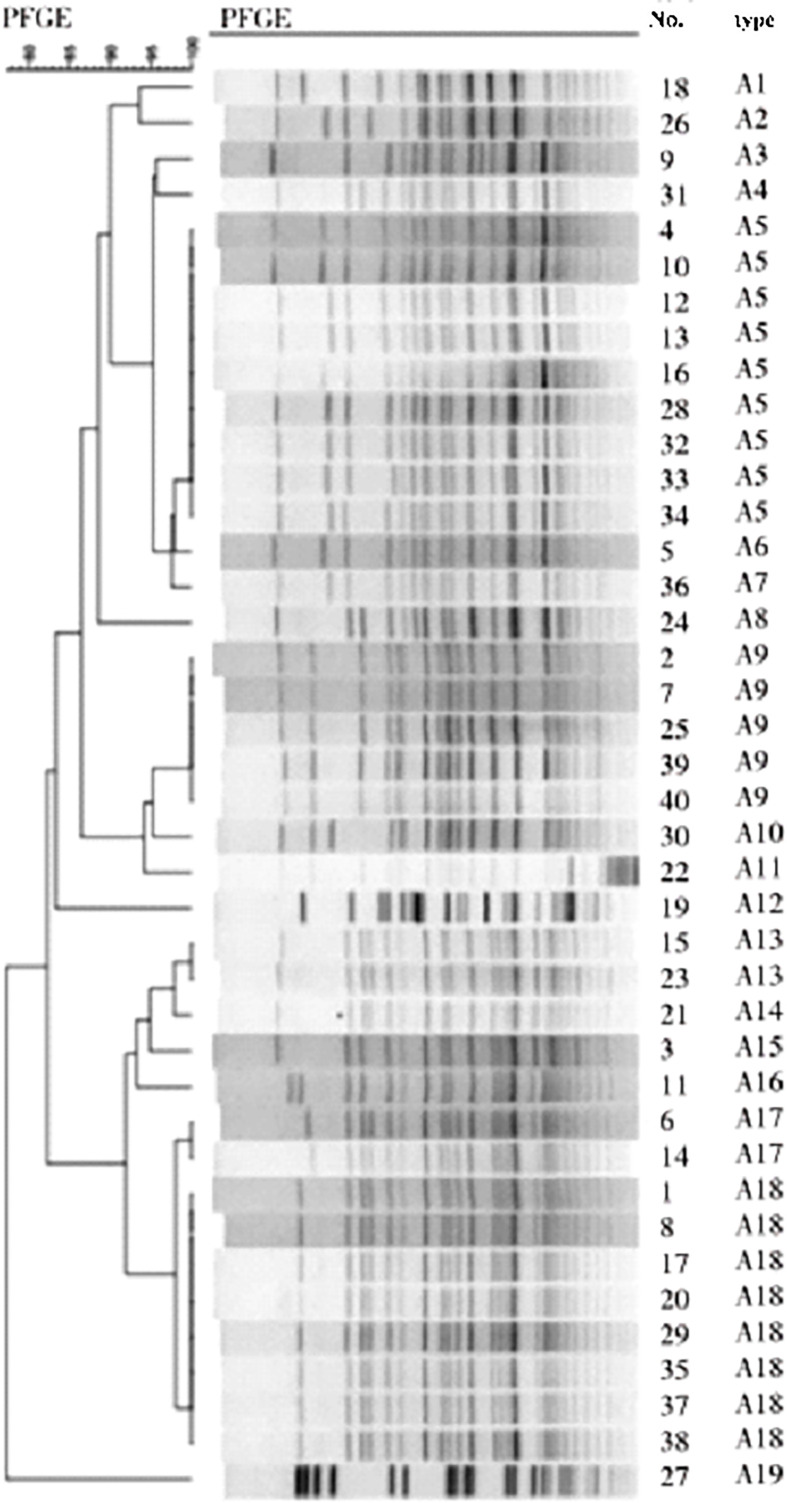
PFGE clustering tree of 40 crabs.

40 CRAB PFGE clustering map showed that the number of electrophoretic bands ranged from 22 to 29, as shown in **Figure 2**. Divided into A1-A19, 19 different types of belt. The resistance spectrum for each type is detailed in **Table 4**. Each band type contained 1-9 strains, of which strains 4, 10, 12, 13, 16, 28, 32, 33, 34 were A5 type, strains 1,8,17,20,29,35,37,38 were A18 type, strains 2,7,25,39,40 were A9 type, strains 15,23 were A13 type, strains 6,14 were A17 type, and the remaining 14 band types contained only 1 strain. See **Figure 3** and **Table 2**.

## Discussion

Acinetobacter baumannii is an aerobic gram-negative conditional pathogen, which widely exists in the hospital environment. Due to its strong viability and resistance, the infection of Acinetobacter baumannii has become a difficult problem to be solved in hospitals all over the world. Due to the irrational use of antibiotics and the lack or inadequate implementation of nosocomial infection management and control measures, the drug resistance of bacteria is on the rise. Multidrug resistant AB has become the main pathogen of nosocomial infection (Anunnatsiri and Tonsawan, 2011) and spread all over the world. Among them, 40 CRAB strains were resistant to cefotaxime, ceftriaxone, levoflOXAcin, tobramycin, ceftazidime, imipenem, meropenem, piperacillin, piperacillin/tazobactam, gentamicin, amikacin, cefepime and minocycline mic50. MIC90 also far exceeded the break point of drug resistance, and the drug resistance phenomenon was very serious. The drug resistance mechanism of multidrug resistant AB is very complex, including the production of various drugs β-Lactamase, decrease of outer membrane permeability, change of drug target, overexpression of efflux pump and production of biofilm (Ming, 2015).

Produced by bacteria β- Lactamases are mainly divided into four categories, and category a mainly refers to broad spectrum β- Lactamases, including TEM, SHV, KPC, etc; Class B metal β- Lactamases, including imp, vim and sim; Class C is amp C enzyme; Class D is OXAcillinase, also known as OXAcarbapenemase, of which OXA-23 is the most common OXAcillinase in China. Various β-The combined action of lactamases can mediate the resistance of Acinetobacter baumannii to penicillin, carbapenems, monocyclic amides and cephalosporins (Wanzhen et al., 2012). In this study, the resistance rate of 40 CRABs to carbapenems and cephalosporins was 100%, indicating that β- Lactamase is one of the causes of drug resistance.

The detection rates of OXA-51 and OXA-23 were 90.0% and 77.5% respectively. The detection rate of OXA-23 gene was basically similar to that reported in the domestic literature (Qinglan et al., 2019), and no new subtype was detected. The appearance of CRAB is closely related to the accumulation of drug-resistant genes by integron. Integron is an original that captures drug-resistant genes and can be transmitted. It exists on chromosomes, plasmids or transposons. Class I ~ III integron is mainly related to ab resistance, of which class I integron is the most common. The detection rate of class I integron (intl1 gene) in our hospital is 72.5%, which is basically similar to that reported in domestic literature. The detection rates of aminoglycoside resistant genes ant (3 ‘‘) -i and AAC (3) -i were 57.5% and 35%, respectively,. 0%. It is reported in the literature that 16S rRNA methylase can cause bacterial resistance to aminoglycoside antibiotics (Daiguihua, Yanbinbin, Wangbin, Fengweisi, Malinlin, Limingcheng, 2019). Such genes can be transmitted between strains through a variety of ways, such as transposons, integrons and plasmids, which may lead to epidemics in hospitals (Yi, 2020), and the epidemics of aminoglycoside resistance genes in different hospitals or in the same hospital at different times (He et al., 2017). The results of this study showed that there were only 11 strains containing aminoglycoside resistant gene ant (3’’) -i + aac(3) -i CRAB, which was not the main resistant gene prevalent in the hospital.

The detection rate of disinfectant resistance gene qacE△1-sull was 80.0%. At present, the most studied disinfectant resistance gene is quaternary ammonium disinfectant resistance gene, namely qac gene. The qac gene family includes qac A, qac B, qac C, qac d, qacE, qac F, etc. (Harding et al., 2018). It is a common phenomenon that clinical AB strains carry qacE△1 gene (Hamidian and Nigro, 2019), which shows resistance through the expression of multiple compound efflux pump genes of pathogenic bacteria. Literature (Lishuwang, Jiangmeijie, Zhanggang, 2013) reported that the detection rate of qacE△1-sull was more than 90%. The detection rate of qacE△1-sull in our hospital was 80%, slightly lower than that reported in the literature, but we should also pay attention to it. The detection rate of integron resistance gene intl1 was 72.5% Integron can make qacE△1-sull gene spread between AB and ab. it is speculated that the high detection rate of qacE△1-sull gene in this hospital may be related to the high detection rate of integron resistant gene. The detection rate of transposon resistance gene in our hospital was 15.0%, which was relatively low. TEM type β- Lactamases belong to broad spectrum β- The encoding gene of lactamase, which is located on the TNL sequence of the transposon of the drug-resistant plasmid, can be transferred to other bacteria through the conjugation of the encoding plasmid, and can hydrolyze the third generation cephalosporins. This is also the main reason for the increasing resistance of AB clinical isolates to cephalosporins in recent years. The detection rate of ADC was as high as 100%, indicating that ADC was an inherent drug resistance gene of ab. Research (Liu et al., 2018) shows that ADC will change its ability to hydrolyze drugs when its coding gene is mutated. The detection rate of conjugated plasmid resistance gene TRAA was 7.5%. No new drug resistance genotype was found in this test.

In this study, the number of CRAB strains is relatively small, and CRAB resistant to imipenem and meropenem has not been studied. Some cases of increased sensitivity of AB to imipenem may be omitted due to the deletion of OXA-23 gene (Scaife et al., 1995). More samples of different types will be collected in the future to analyze the relationship between drug resistance and drug resistance genes and the homology. KPC gene is not detected in this study, so KPC chromogenic plate method can be considered for verification.

## Conclusion

This study showed that the main drug resistance genes of CRAB were ADC, OXA-51 and OXA-23. TEM, ADC, OXA-51 and OXA-23 genotypes are higher in CRAB, which is the main cause of β-lactam resistance. Homology study showed that there was a small range of drug resistance gene clone transmission in Acinetobacter baumannii in our hospital. Hospital infection control monitoring should be actively carried out to prevent CRAB clone transmission. We should reasonably use antibacterial drugs, strengthen the improvement of existing antibacterial drugs and the research and development of new antibacterial drugs.

## Data availability statement

The original contributions presented in the study are included in the article/supplementary material. Further inquiries can be directed to the corresponding author.

## Ethics statement

This study was conducted in accordance with the Declaration of Helsinki and approved by the ethics committee of Inner Mongolia Baogang hospital.

## Author contributions

H-BG conceived of the study, and H-LH participated in its design and coordination, and Y-YL helped to draft the manuscript. All authors read and approved the final manuscript.
