# Corrigendum: Detection and homology analysis of carbapenem resistant Acinetobacter baumannii resistance gene

**DOI:** 10.3389/fcimb.2023.1141959

**Published:** 2023-03-24

**Authors:** Hua-Liang Huang, Yue-Yu Li, Hong-Bo Guo

**Affiliations:** Department of Laboratory, Inner Mongolia Baogang Hospital, Baotou, China

**Keywords:** carbapenem resistant Acinetobacter baumannii, drug resistance gene, pulsed field gelelectrophoresis, homology, infection microbiology

## Error in author list

In the published article, there was an error in the author list as the order of the authors was incorrect. The corrected author list appears below.

Hua-Liang Huang^†^, Yue-Yu Li^†^, Hong-Bo Guo^*^

The authors apologize for this error and state that this does not change the scientific conclusions of the article in any way. The original article has been updated.

